# State Medical Association Matters

**Published:** 1904-11

**Authors:** 


### State Medical Association Matters.

The State Medical Association's Committee on Public Policy and Legislation met at the Oriental Hotel, Dallas, November 5th inst., pursuant to call of the President. The following proceedings are reported by the Secretary:

The Committee on Public Policy and Legislation of the State Medical Association of Texas met on November 5th at Dallas. There were present the entire committee: Drs. Daniel, Wilson, Cantrell, Bennett and Chase. The following resolutions, after discussion, were adopted:

1. Resolved, That this committee deems it unwise to attempt at the approaching session of the Legislature to secure any legislation looking toward the establishment of a State Board of Health, owing to the lack of definite instructions from the House of Delegates; believing it to be more expedient that such a bill shall be presented for consideration and ratification by the medical profession of the State at the next regular meeting of the House of Delegates (at Houston, April, 1905), and for other reasons, all of which have been duly considered.

2. Resolved, That this committee shall work faithfully at the ensuing session of the Legislature for the passage of the following amendments to the practice act, which were recommended by the Board of Medical Examiners and adopted by the House of Delegates at Austin, April, 1904:

Sec. 4. Amend by adding to the last line after the words "surgery and midwifery," the following: "The said Boards shall each procure a seal which shall be the official seal of the Boards."

Sec. 5. Amend by striking out the words "the seal of the State of Texas," in the last line, and by adding instead "the seal of the Board granting such license."

Sec. 6. Amend by adding in line 22, after the word "character," "and that he or she is a graduate of a reputable medical school of recognized standing, requiring for graduation attendance upon not less than three regular sessions of not less than six months each, in three separate calendar years."

Sec. 8. Amend by adding in line 22, after the words "receive a license from the Board of Medical Examiners of Texas to practice in this State," the following: "Provided, that the medical laws and examining boards of such States grant equal rights and recognition to the licentiates of the boards herein created, and provided that the applicant has not previously been rejected by one of the boards of this State."

Sec. 8. Amend by adding after the word "midwifery," at the end of the section, the following: "Sixth, provided that all persons changing their residence to another county shall have their diplomas or licenses registered with the district clerk of said county with certificate of date of issue and previous record."

Sec. 13. Amend by adding in the fourth line, after the word "imprisonment," the following: "In the county jail," and by striking out in the seventh line in the same section (13), after the words "surgeon or midwife," the following sentence: "Provided, that the provisions of this act do not apply to persons treating disease who do not prescribe or give drugs or medicine," and add instead the following sentence: "Provided, that those persons treating disease who do not prescribe or give drugs or medicine shall be examined in all the branches provided for in this act, except chemistry, materia medica and therapeutics."

3. Resolved, That this committee recommend that Dr. Russ, Chairman of Board of Councilors, be requested to call a meeting of the Councilors to confer with delegates from county societies on November 14th concerning a bill for a board of health, and other matters of importance to medical organization.

4. Resolved, That this committee favor the proposed bill of Judge Terrell providing for civil service reform, looking to more permanent tenure of office in our eleemosynary institutions, and that the co-operation of the entire profession be recommended.

5. Resolved, That Drs. Daniel and Bennett, both of Austin, be appointed an Executive Committee of the Committee on Public Policy and Legislation to keep this committee as well as Councilors and county secretaries informed of the progress of legislation, and by letters and telegrams to direct the entire Association in ways and means of influencing local representatives to favor desired medical legislation.

6. Resolved, That a copy of these resolutions be sent immediately to the secretary of each county society and to each Councilor.

I. C. CHASE, Secretary. F. E. DANIEL, President.

The only other legislation for which the committee was instructed by the House of Delegates to ask was the passage of an act legalizing dissection, the anatomical bill drawn by Professor Keiller of the Medical Department of the University of Texas and which was presented but not acted on at last session. A knowledge of anatomy is required by Texas law, but it is a felony to secure and dissect a body. Hence the efforts of the committee will be limited this session to securing the above amendments and the passage of the anatomy act.

---

This issue being delayed, I can give briefly an account of what was done at the meeting of the Committee on Public Policy and Legislation in joint conference with representatives from the Committee on Legislation from various county medical societies and the Board of Councilors, referred to elsewhere.

In response to the call of the President, there assembled at Austin, November 14th inst., a large representation of county societies and a majority of the general committee and Councilors. The meeting was characterized by interest and enthusiasm, and the work for which the conference was called was completed in a two days' session in a most satisfactory manner. An anatomical (dissecting) bill was perfected and unanimously approved, as were a number of amendments to the practice act (published elsewhere in this issue), and these two bills having received the sanction of the House of Delegates at the Austin meeting last April will be presented to the approaching Legislature, and pushed.

A bill for a State Board of Health was perfected and approved without a dissenting vote. It will now be acted upon by the general Committtee on Public Policy and Legislation, printed and sent to each county medical society for ratification and endorsement. Then it will be submitted to the House of Delegates and if approved its passage will be asked at the hands of the Thirtieth Legislature, January, 1907. Meantime the members of the medical profession will do missionary work and prove a factor in the election of the next administration.

The conference endorsed the Hepburn Pure Food bill that will go before the United State Senate next month, and the Secretary was instructed to write to the Texas Senators that it is the sense of the Texas profession, as represented in this conference, that the bill should become a law, and ask their support of same.

Thus the State Medical Association of Texas will go before the Legislature a united body, unanimously asking for legislation in the interest of the public health, and we are assured, when we do that, the legislation will be forthcoming.

Fuller details, with the full text of these bills, will appear in our December number.

Every Texas physician in sympathy with this work, and who would keep in touch with the profession, should at once subscribe to the JOURNAL.

---

JOINT CONFERENCE OF LEGISLATIVE COMMITTEES AT AUSTIN.—A meeting of the State Medical Association Committee on Public Policy and Legislation has been called for November 14th and 15th at Austin, for the purpose of taking action upon the draft of a proposed bill for a State Board of Health, amendments to the medical practice act, an anatomical bill and other matters. Any bill for a State Board of Health (or any other purpose) must be submitted by the committee to the House of Delegates and approved before it can go to the Legislature, and the House of Delegates will not meet till April. Representatives from every county medical society in the State have been invited to meet with the committee for a general conference and discussion, with a view to ascertaining the sentiment and wishes of the entire regular profession of the State, and, as this goes to the printers, the conference is in session. A full report of its action, together with the State Board of Health bill agreed upon (if any is agreed upon), will be published in the December number of the JOURNAL.

There is being held at the same time and place a meeting of the fifteen Councilors of the fifteen medical ditsricts of the State, who will push the matter of organization of the several district societies, and take steps to increase the membership of county medical societies, until every reputable physician is enrolled in the State. At present 47.7 per cent are so enrolled.

